# Adverse Effects Related to Paediatric Influenza Vaccination and Its Influence on Vaccination Acceptability. The FLUTETRA Study: A Survey Conducted in the Region of Murcia, Spain

**DOI:** 10.1111/irv.13331

**Published:** 2024-06-21

**Authors:** Matilde Zornoza Moreno, Jaime Jesús Pérez Martín, María Cruz Gómez Moreno, María del Carmen Valcárcel Gómez, Marta Pérez Martínez, Francisca Isabel Tornel Miñarro

**Affiliations:** ^1^ Prevention and Health Protection Service Ministry of Health Murcia Spain

**Keywords:** adverse effects, influenza vaccines, paediatric vaccination, parents' acceptance

## Abstract

**Background:**

During the 2022–23 season, three autonomous communities recommended influenza vaccination for all children between 6 and 59 months. The objective is to evaluate the adverse effects associated with the administered influenza vaccines in the Region of Murcia, as well as their influence on the recommendation of the same to acquaintances or repetition in future seasons.

**Material and Methods:**

Cross‐sectional descriptive study with an online questionnaire sent to the parents of vaccinated minors of 6–23 months of age receiving inactivated intramuscular vaccine (IIV) or 24–59 months of age receiving live‐attenuated intranasal vaccine (LAIV).

**Results:**

Among 4971 surveys received, the most common adverse effect for LAIV and IIV was runny nose (40.90%) and local pain (31.94%), respectively. Sixty percent of adverse effects lasted ≤ 1 day, and around 10% lasted ≥ 3 days. The interference of adverse effects with the minor's daily life was very infrequent (3.32%), as was the need for visiting the medical office (2.68%). Overall, 96.44% of parents would recommend influenza vaccination to friends and relatives after the experience. Only 3.56% would not recommend it, while 1.68% would not vaccinate their child against influenza again. The most frequently cited reason being adverse effects.

**Conclusions:**

Our study shows the safety of influenza vaccines. Despite the low impact of adverse effects, they influence some parents in their intention to continue vaccinating or recommending it to acquaintances, which remarks the need to reinforce the information given to parents so that this fact does not influence decision‐making.

## 1 Introduction

Influenza is a viral infection that every season poses a public health problem, with cases of greater severity being concentrated among high‐risk groups, such as the elderly (60 years of age or older), children under 5 years of age, pregnant women and people with certain chronic illnesses [1].

In 2012, the World Health Organization included children aged 6 to 59 months as a target population for influenza vaccination due to the high disease burden in this age group [2]. That same year, the European Centre for Disease Prevention and Control issued a technical report favouring vaccination in this age group [3]. Today, more than 70 countries have incorporated it in their childhood and adolescence vaccination schedules [4].

In Spain, the greatest disease burden occurs in the group under 5 years of age. In addition, 68.4% of hospitalizations classified as serious and 56.1% of admissions to intensive care between 2013–2014 and 2019–2020 occurred in children with no known pathology of risk [5]. With regard to mortality, 83% of the children who died had been hospitalized in serious condition and had one or more risk factors. Considering the FluMOMO model, an average of 28 deaths in children under 5 years of age attributed to influenza was estimated between 2013 and 2017, reaching 56 deaths in 2017 [6].

Due to this disease burden, in Spain, in 2022, the Public Health Commission approved the document ‘Recommendations for influenza vaccination in children aged 6 to 59 months’ [7]. Three autonomous communities, Andalusia, Galicia and the Region of Murcia, started universal vaccination in this age group as early as the 2022–2023 season. In our region, the intranasal live attenuated influenza vaccine (LAIV) was administered to children under 2 to 4 years of age in this campaign, while the intramuscular inactivated influenza vaccine (IIV) was used in children 6–23 months of age [8]. The Spanish Association of Pediatrics preferentially recommends the intranasal influenza vaccine as of 2 years of age in its immunization calendar for the year 2023 [9].

Both the LAIV and IIV have a good safety profile. As described in the Summary of Product Characteristics, the most common adverse reaction with LAIV is nasal congestion/rhinorrhoea [10], while with IIV, the most common local adverse effect was pain at the administration site and irritability among the systemic adverse effects [11].

Therefore, in this first influenza vaccination campaign for the paediatric population aged 6 to 59 months in 2022–2023, the primary objective was to monitor the presence of adverse events after the administration of influenza vaccines. The proposed secondary objectives are to also separately assess the adverse events associated with each of the vaccines administered and their influence on a recommendation for influenza vaccination to friends and family.

## 2 Material and Methods

### 2.1 Study Framework

This analysis was extracted from the FLUTETRA study, a cross‐sectional descriptive study outlining the outcomes of the first universal influenza vaccination campaign (2022–2023) in the paediatric population aged 6 to 59 months in the Region of Murcia. In this work we present the safety data obtained from the FLUTETRA study based on the adverse events associated with paediatric influenza vaccination. On day 7 after vaccination, a single text message with a link to an electronic data collection form was sent to the children's parent/legal guardian, using the contact mobile phone number available in the children's file.

The following inclusion criteria were considered for participation: children aged 6–23 months vaccinated with IIV and children aged 24–59 months vaccinated with LAIV in the Region of Murcia. Children aged 6–59 months whose parents/legal guardians do not gave their consent to participate were excluded.

A questionnaire was designed to collect information on adverse events associated with influenza vaccination in the 7 days following the influenza vaccination, together with socio‐demographic information of vaccinated children as well as of their parents/legal guardians.

### 2.2 Study Variables

After agreeing to participate in the study, the parents/legal guardians completed the questionnaire (see Data S1) submitted, from which information was obtained on sociodemographic data from children and parent, data related to the occurring adverse effects, co‐administration with other vaccines and the influence of the onset of adverse effects on their likelihood to recommend the influenza vaccine to others.

### 2.3 Statistical Analysis

Statistical analysis was performed using SAS v9.4 software under the SAS Enterprise Guide v8.3 interface.

For the descriptive analysis of qualitative variables, frequency and percent distribution tables were made. To assess the comparison between qualitative variables, a chi‐squared test or a Fisher's exact test was performed, depending on the application criteria of each.

### 2.4 Ethics

The study was approved by the Medicinal Product Research Ethics Committee of Area1 of the Region of Murcia, where the study was conducted. Participants received information and accepted their participation prior to completing the questionnaire.

This study has complied with the standards of Good Clinical Practice and the regulations and recommendations appearing in the Declaration of Helsinki, as set down in the current legislation on Biomedical Research.

## 3 Results

By the end of the study, on 1 February 2023, a text message with the data collection form was sent to the parents of 15,539 minors vaccinated with LAIV and 7326 minors vaccinated with IIV with a registered contact mobile phone number available. The response rate was 21.74% (N = 4971).

Statistically significant differences were found with respect to the proportion of first‐born infants regarding each type of vaccine (*p* < 0.001). There is a significantly higher proportion of children with respiratory and allergic disease in the group vaccinated with LAIV (older), accounting for 44.16% of all children with disease (24.07% of those vaccinated with IIV and 62.09% of those vaccinated with LAIV). No statistically significant differences were found due to a history of prematurity (*p* = 0.154). Table 1 shows the rest of the sociodemographic variables.

**TABLE 1:** Sociodemographic variables of the children vaccinated in the 2022–2023 influenza vaccination campaign for the paediatric population aged 6–59 months in the study's autonomous community and of their parents/legal guardians who have completed the electronic registry form.

		IIV *N* (%)	LAIV *N* (%)	Overall *N* (%)	*p*
Age of the minor	6–11 months	499 (34.77%)	0 (0.00%)	499 (10.05%)	< 0.001
	1 year	936 (65.23%)	0 (0.00%)	936 (18.84%)	
	2 years	0 (0.00%)	1176 (33.31%)	1176 (23.68%)	
	3 years	0 (0.00%)	1173 (33.22%)	1173 (23.62%)	
	4 years	0 (0.00%)	1182 (33.47%)	1182 (23.83%)	
Sex of the child	Male	737 (51.54%)	1830 (51.87%)	2391 (48.23%)	0.832
	Female	693 (48.46%)	1698 (48.13%)	2567 (51.77%)	
Minor's number of siblings	0	729 (50.94%)	1226 (34.79%)	1955 (39.46%)	< 0.001
	1	533 (37.25%)	1768 (50.17%)	2301 (46.44%)	
	2	134 (9.36%)	421 (11.95%)	555 (11.20%)	
	> 2	35 (2.45%)	109 (3.09%)	144 (2.91%)	
Minor's history of chronic disease	Yes	42 (2.93%)	177 (5.03%)	219 (4.42%)	0.001
	No	1392 (97.07%)	3343 (94.97%)	4735 (95.58%)	
History of prematurity	Yes	114 (7.98%)	313 (8.93%)	427 (8.66%)	0.282
	No	1314 (92.02%)	3191 (91.07%)	4505 (91.34%)	
Parent/legal guardian age	< 20 years	19 (1.32%)	11 (0.31%)	30 (0.60%)	< 0.001
	20–29 years	159 (11.05%)	201 (5.69%)	360 (7.24%)	
	30–39 years	987 (68.59%)	2071 (58.64%)	3058 (61.52%)	
	40–49 years	277 (19.25%)	1224 (34.65%)	1501 (30.20%)	
	≥ 50 years	2 (0.14%)	28 (0.79%)	30 (0.60%)	
Parent/legal guardian gender	Male	131 (9.14%)	249 (7.07%)	380 (7.47%)	0.013
	Female	1302 (90.86%)	3272 (92.93%)	4574 (92.53%)	
Parent/legal guardian birth place	Spain	1300 (91.16%)	3183 (91.13%)	4483 (90.89%)	< 0.001
	WHO European Region	29 (2.03%)	54 (1.54%)	83 (1.68%)	
	WHO Eastern Mediterranean Region	7 (0.49%)	19 (0.54%)	26 (0.53%)	
	WHO Region of the Americas (South)	84 (5.89%)	224 (6.04%)	318 (6.45%)	
	WHO Region of the Americas (North)	4 (0.28%)	5 (0.14%)	9 (0.18%)	
	WHO African Region	2 (0.14%)	7 (0.20%)	9 (0.18%)	
	WHO Western Pacific Region	0 (0.00%)	2 (0.06%)	2 (0.04%)	
	WHO South‐East Asian Region	0 (0.00%)	2 (0.06%)	2 (0.04%)	
Parent/legal guardian level of education	No formal education	7 (0.49%)	19 (0.54%)	26 (0.52%)	0.007
	Primary education	73 (5.09%)	259 (7.35%)	332 (6.70%)	
	Secondary studies	487 (33.96%)	1265 (35.91%)	1752 (35.34%)	
	University studies	867 (60.46%)	1980 (56.20%)	2847 (57.43%)	
Parent/legal guardian history of chronic disease	Yes	259 (18.06%)	656 (18.62%)	915 (18.46%)	0.645
	No	1175 (81.94%)	2867 (81.38%)	4042 (81.54%)	

**FIGURE 1:**
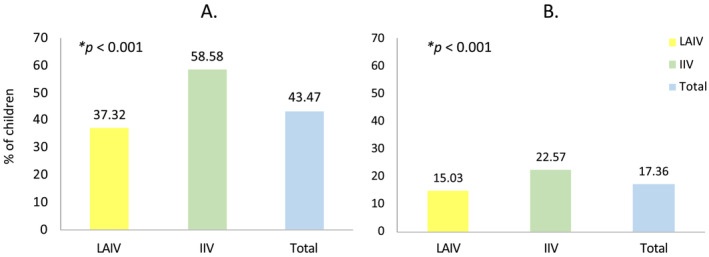
Onset of adverse effects related to the influenza vaccination. (A) Percentage of children presenting at least one adverse effect, overall and by type of vaccine received. (B) Percentage of children who required any treatment among those who have been vaccinated, overall and by type of vaccine received.

**FIGURE 2:**
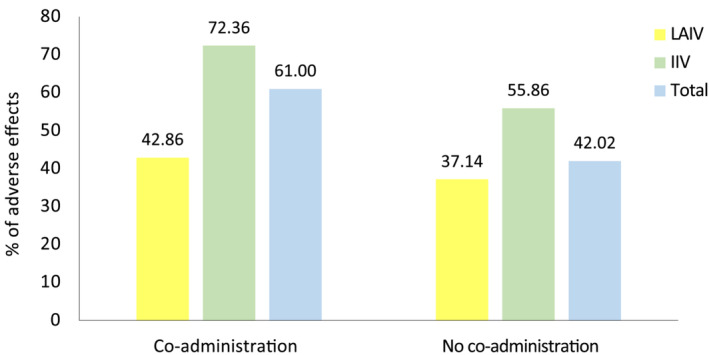
Comparison of the percentages of adverse effects based on the influenza vaccine co‐administration with another vaccine or not, overall (blue colour) and by type of vaccine (LAIV yellow and IIV green).

As shown in Figure 1, 43.47% of children had at least one adverse effect within 7 days after influenza vaccination, with IIV being significantly more reactogenic than LAIV (*p* < 0.001). No undescribed nor anaphylactic adverse effects were reported. A total of 17.36% indicated that their children had required any treatment, and this percentage was significantly higher in those vaccinated with IIV (*p* < 0.001). Vaccine co‐administration increased reactogenicity overall (*p* < 0.001), as shown in Figure 2, with an increase of about 19%.

**FIGURE 3:**
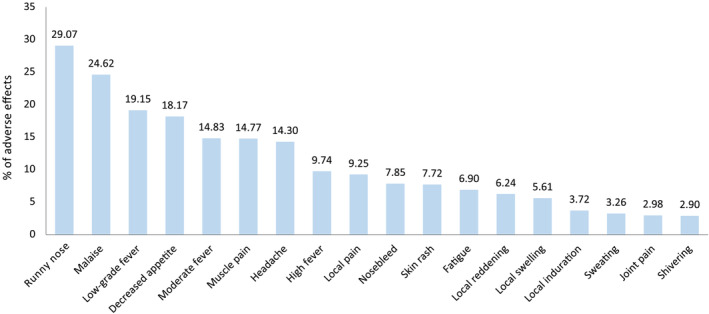
Frequency of occurrence of each adverse effect among the total sample of children vaccinated with either product during the 2022–2023 campaign.

**FIGURE 4:**
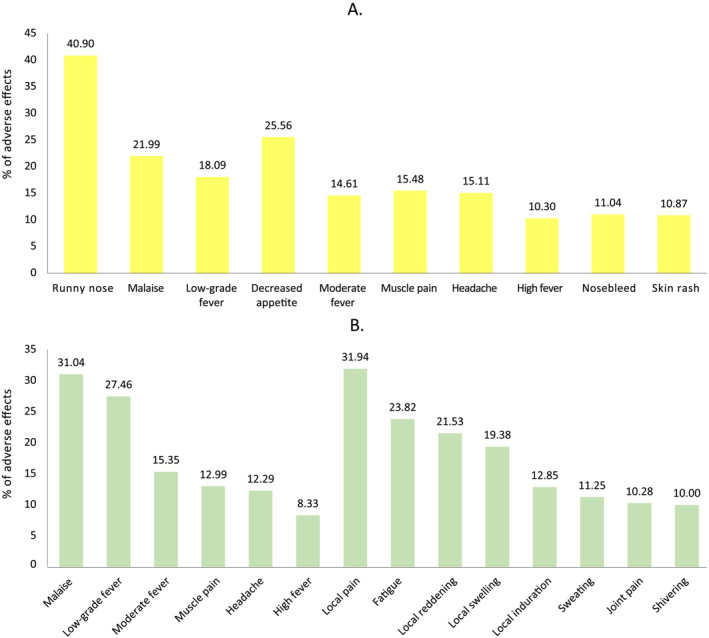
Frequency of occurrence of each of the adverse effects based on the vaccine received. (A) Vaccinated with LAIV (yellow). (B) Vaccinated with IIV (green).

The most commonly reported adverse effect was runny nose, accounting for more than 29%. The frequency of the remaining adverse effects appearing overall and stratified by vaccine type are displayed in Figures 3 and 4, respectively.

**FIGURE 5:**
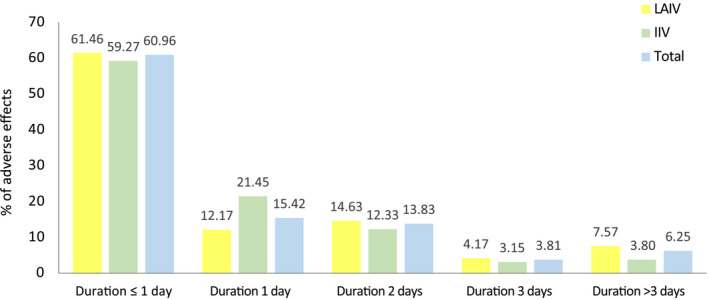
Duration of each adverse effect recorded, both overall (blue) and by type of vaccine received (LAIV yellow and IIV green).

The adverse effects recorded were mostly short‐term (Figure 5), lasting ≤ 1 day in more than 60% of cases. The longest lasting adverse effects, ≥ 3 days, accounted for about 10%. Considering the duration of the most common adverse effects per vaccine, the most common with LAIV was runny nose, lasting ≤ 1 day in 48.16% of cases and only > 3 days in 15.57%; compared to local pain related to IIV, in 85.43% of cases, its duration was ≤ 1 day, and in 0.6% of cases, it lasted > 3 days.

Interference of the adverse effects with the daily life of the minor was very uncommon (3.32% and 3.65% with LAIV and 2.72% with IIV). Medical consultation was required in very few cases (2.68% and 3.17% with LAIV and 1.77% with IIV).

Most parents or legal representatives would recommend vaccination with IIV (96.36%) and LAIV (96.47%) to relatives and friends. Only 3.56% would not recommend influenza vaccination after their child's experience (3.65% with IIV and 3.53% with LAIV; *p* = 0.844), while 1.68% of parents would not revaccinate their child against influenza next season (1.33% with IIV and 1.82% with LAIV; *p* = 0.022). When those people who responded negatively were asked for the reasons, despite their low frequency, the most common response was the onset of adverse effects, 51.51% in the those vaccinated with IIV cited them as the reason (2.36%), while the percentage was lower for those vaccinated with LAIV, 43.55% (2.01% of the total).

## 4 Discussion

As regards the proportion of children with underlying disease in each group, those receiving LAIV are older and have therefore had a longer time to onset of chronic pathologies, such as respiratory or allergic diseases (those most commonly reported), which are much less common in the first months of life. Moreover, we should consider the hypothesis that healthy children are vaccinated less, thus it is likely to find a higher proportion of children with disease among the older children vaccinated with LAIV.

With regard to the nationality of parents who responded to the survey (90.78% Spanish and the remaining nationalities are registered in Table 1), these data differ from the total number of vaccinated in the 2022–2023 campaign, where parents from Spain accounted for 78.5%. In relation to other common immigrant populations in our region, such as those from the Eastern Mediterranean Region (0.53% of responders and 4.46% of subjects vaccinated) or the South American region (6.45% of responders and 8.21% of total vaccinated subjects), differences were in fact observed. Though this could involve sample selection bias, since some parents could have potentially not responded due to language barriers or a lesser interest, no greater vaccine reactogenicity based on parent origin has been reported in the literature. Only an increased cumulative risk of febrile crisis in the first 5 years of life has been reported, ranging from 2%–5% in children from North America and Europe to 6%–9% in those of Asian origin [12]; however, no febrile seizures associated with influenza vaccination have been reported in our sample, so differences in parental origin with respect to the total number of vaccinated subjects should not influence the results.

This study confirms the safety of both vaccines administered in the 2022–2023 influenza vaccination campaign for the paediatric population aged 6 to 59 months. For those children vaccinated with LAIV, the most reported adverse effect, as in previous studies [13, 14], were respiratory symptoms, such as runny nose/rhinorrhoea (40.90%), but less frequently found than in studies such as Gasparini et al. (52.5%) [14] or Carter et al. (just under 60%) [13]. Other adverse effects with a higher frequency of occurrence were decreased appetite and malaise, with fever or irritability appearing in second place in these other studies [13, 14].

Based on their duration, as with the studies described above, the adverse effects related to the administration of LAIV are mainly mild, with 60% of them lasting less than 1 day and 10% lasting more than 3 days. Families were asked about their interference with the lives of the children or the need to consult a healthcare professional, which could mean that they were more significant adverse effects, which occurred in both cases in less than 4%, which is also consistent with previous reports in the literature, such as that of Gasparini et al. [14]. Just over 10% required treatment for the adverse effects presented, which is low in frequency.

We observed that the occurrence of adverse effects related to vaccination is significantly more frequent (58.58%) in IIV‐vaccinated children than in those vaccinated with LAIV (37.52%). A similar study reported that the most common adverse effects observed after LAIV vaccination were local, and malaise was the most common among systemic adverse events. However, in our study, the frequency of such episodes (both local and systemic) was lower. A retrospective cohort study in the United Kingdom using a large database (over 1 M patients, all ages) published that the most common adverse effects observed after LAIV vaccination were also local but reported higher incidence of fever in comparison to IIV, which was not observed in our study [15]. Additionally, the work of de Lusignan et al. also reported a lower incidence of AE of interest in UK patients (all ages) vaccinated with LAIV (22%) than in patients vaccinated with trivalent or quadrivalent vaccines [16].

Despite the frequency of occurrence of adverse effects with IIV, they are usually minor, with less than 10% being grade 3 [17]. In our case, it is also shown that most adverse effects are of low severity, considering that we report a low percentage of adverse effects lasting 3 days or more (6.95%), that interfere with the life of the vaccinated patient (2.72%) or that require consultation with a healthcare professional (1.77%).

Few studies have evaluated the frequency of adverse effects based on the co‐administration or not of influenza vaccines with other vaccines. However, in patients co‐administered LAIV with Measles, Mumps and Rubella (MMR) vaccine [18] or oral polio vaccine [19], there was a greater frequency of adverse effects in those who received them jointly, as occurred in our study, in which the co‐administration group had a 5% greater incidence of adverse effects. With regard to the co‐administration of IIV with other vaccines, there are discordant results in the different studies, some of which do not associate a lower frequency of fever between concomitant or sequential administration of IIV with other vaccines administered in the paediatric population, such as 13‐valent pneumococcal conjugate vaccine or DTP vaccine [20], while others suggest a greater risk of other adverse effects such as febrile seizures if IIV is co‐administered with the pneumococcal vaccine [21]. Our study showed that the co‐administration of IIV together with another vaccine presented 17% more adverse effects. Compared to both vaccines, we found 29.5% more adverse effects in IIV co‐administration as compared to LAIV, while in the case of vaccination alone, the difference decreased to 18.72%, meaning LAIV behaves better when co‐administered with other vaccines.

Despite the low frequency, the occurrence of adverse effects related to influenza vaccination, as in other previous studies [14], is the factor that most influences the decision of parents not to recommend it to family and friends or not to repeat influenza vaccination in a subsequent season, with very slight differences by type of vaccine. However, this makes it a critical matter for us to take into consideration in upcoming campaigns to inform the population of the frequency of onset of these adverse effects and to communicate that the benefit of vaccination is greater than its possible inconveniences, so that it does not influence the decision to continue vaccinating their children in the following seasons or their likelihood to recommend vaccination to friends or relatives.

As other limitations of the study, our response rate (21.74%) is somewhat lower than similar studies, such as Gasparini et al., with a survey completion rate of 34.72% of vaccinated children [14], so parents of children with more adverse effects may have responded to a greater extent due to increased awareness. Although we do not have the information on the level of education of all unvaccinated children's parents, 57.46% of the subjects in our sample had a university education (lower than the data obtained in other studies, with 69.6% [14]). As a result, the influence of this parameter would be low or very low.

It should be concluded that although more than 40% of vaccinated children had adverse effects related to the vaccine, these were mostly short in duration, did not require consultation with a healthcare professional or medical treatment and did not interfere with their daily life, both overall and by type of vaccine. The most common adverse effects were runny nose for those vaccinated with LAIV and local pain for those vaccinated with IIV, which thereby reiterate their safety. Despite the low frequency, the onset of adverse effects does influence a certain percentage of parents in their intention to continue vaccinating their children or to recommend it to family and friends, which makes it essential that we reinforce the information given to them so that this fact does not ultimately influence their decision, given the importance of vaccination.

## Author Contributions

**Matilde Zornoza Moreno:** conceptualization, formal analysis, writing–original draft, writing–review and editing. **Jaime Jesús Pérez Martín:** conceptualization, formal analysis, writing–original draft, writing–review and editing. **María Cruz Gómez Moreno:** formal analysis, writing–original draft, writing–review and editing. **María del Carmen Valcárcel Gómez:** formal analysis, writing–original draft, writing–review and editing. **Marta Pérez Martínez:** formal analysis, writing–original draft, writing–review and editing. **Francisca Isabel Tornel Miñarro:** formal analysis, writing–original draft, writing–review and editing.

## Ethics Statement

The study was approved by the Medicinal Product Research Ethics Committee of Area1 of the Region of Murcia. All participants received appropriate and sufficient information and accepted their participation prior to completing the questionnaire.

## Consent

All participants received appropriate and sufficient information and accepted their participation prior to completing the questionnaire.

## Conflicts of Interest

M.Z.M. and J.J.P.M. declare having received funding from AstraZeneca for training and dissemination activities. J.J.P.M. declares having received funding from CSL Seqirus for training and dissemination activities and M.Z.M., M.C.G.M., M.C.V.G., M.P.M. and F.I.T.M. for training activities.

## Supporting information


**Data S1** Surveys conducted on parents/legal guardians of children vaccinated with each one of the vaccines.

## Data Availability

The data that support the findings of this study are available on request from the corresponding author. The data are not publicly available due to privacy or ethical restrictions.
