# Supplementary material for: Adverse Effects Related to Paediatric Influenza Vaccination and Its Influence on Vaccination Acceptability. The FLUTETRA Study: A Survey Conducted in the Region of Murcia, Spain
Source: Influenza Other Respir Viruses. 2024 Jun 21;18(6):e13331. doi: 10.1111/irv.13331 (PMC11190946; doi:10.1111/irv.13331)
Supplement: Supplementary file 1 — Data S1 Surveys conducted on parents/legal guardians of children vaccinated with each one of the vaccines. [file IRV-18-e13331-s001.docx]

**Supplementary file 1. Surveys conducted on parents/legal guardians of children vaccinated with each one of the vaccines.**

The original text in the survey was in Spanish (from Spain). It has been translated for publication.

Survey of parents/legal guardians of children 6 to 23 months of age vaccinated with IIV:

1. How old is your child? 6-11 months/1 year.
2. Is the child a boy or girl? Girl/Boy.
3. Where was your child born? Spain/Fill in free text.
4. Was your child born prematurely? Yes/No.
5. How many brothers or sisters does your child have (excluding the child)? None/1 sibling/2 siblings/>2 siblings.
6. Does your child have any chronic illnesses? Yes/No.
7. If yes, please specify what type.
8. How old are you? Under 20 years/20-29 years/30-39 years/40-49 years/Over 50 years.
9. What is your gender? Male/Female.
10. What is your country of birth? Spain/Fill in free text.
11. What is your level of education? No studies/Primary studies/Secondary studies/University studies.
12. Do you have any chronic illnesses? Yes/No.
13. If yes, please specify what type.
14. Did your child receive any more vaccines on the same day he/she received the flu vaccine? Yes/No.
15. Please mark which of the following adverse effects you observed after the administration of the vaccine [Headache]: Duration <1 day/Duration 1 day/Duration 2 days/Duration 3 days/Duration >3 days/It interfered with the child’s usual activity/It did not interfere with the child’s usual activity/A phone or face-to-face consultation with his/her paediatrician regarding the adverse effect was required/No phone or face-to-face consultation with his/her paediatrician regarding the adverse effect was required.
16. Please mark which of the following adverse effects you observed after the administration of the vaccine [Pain at the injection site]: Duration <1 day/Duration 1 day/Duration 2 days/Duration 3 days/Duration >3 days/It interfered with the child’s usual activity/It did not interfere with the child’s usual activity/A phone or face-to-face consultation with his/her paediatrician regarding the adverse effect was required/No phone or face-to-face consultation with his/her paediatrician regarding the adverse effect was required.
17. Please mark which of the following adverse effects you observed after the administration of the vaccine [Reddening at the injection site]: Duration <1 day/Duration 1 day/Duration 2 days/Duration 3 days/Duration >3 days/It interfered with the child’s usual activity/It did not interfere with the child’s usual activity/A phone or face-to-face consultation with his/her paediatrician regarding the adverse effect was required/No phone or face-to-face consultation with his/her paediatrician regarding the adverse effect was required.
18. Please mark which of the following adverse effects you observed after the administration of the vaccine [Swelling at the injection site]: Duration <1 day/Duration 1 day/Duration 2 days/Duration 3 days/Duration >3 days/It interfered with the child’s usual activity/It did not interfere with the child’s usual activity/A phone or face-to-face consultation with his/her paediatrician regarding the adverse effect was required/No phone or face-to-face consultation with his/her paediatrician regarding the adverse effect was required.
19. Please mark which of the following adverse effects you observed after the administration of the vaccine [Induration at the injection site]: Duration <1 day/Duration 1 day/Duration 2 days/Duration 3 days/Duration >3 days/It interfered with the child’s usual activity/It did not interfere with the child’s usual activity/A phone or face-to-face consultation with his/her paediatrician regarding the adverse effect was required/No phone or face-to-face consultation with his/her paediatrician regarding the adverse effect was required.
20. Please mark which of the following adverse effects you observed after the administration of the vaccine [Fatigue]: Duration <1 day/Duration 1 day/Duration 2 days/Duration 3 days/Duration >3 days/It interfered with the child’s usual activity/It did not interfere with the child’s usual activity/A phone or face-to-face consultation with his/her paediatrician regarding the adverse effect was required/No phone or face-to-face consultation with his/her paediatrician regarding the adverse effect was required.
21. Please mark which of the following adverse effects you observed after the administration of the vaccine [Muscle pain]: Duration <1 day/Duration 1 day/Duration 2 days/Duration 3 days/Duration >3 days/It interfered with the child’s usual activity/It did not interfere with the child’s usual activity/A phone or face-to-face consultation with his/her paediatrician regarding the adverse effect was required/No phone or face-to-face consultation with his/her paediatrician regarding the adverse effect was required.
22. Please mark which of the following adverse effects you observed after the administration of the vaccine [Joint pain]: Duration <1 day/Duration 1 day/Duration 2 days/Duration 3 days/Duration >3 days/It interfered with the child’s usual activity/It did not interfere with the child’s usual activity/A phone or face-to-face consultation with his/her pediatrician regarding the adverse effect was required/No phone or face-to-face consultation with his/her pediatrician regarding the adverse effect was required.
23. Please mark which of the following adverse effects you observed after the administration of the vaccine [Sweating]: Duration <1 day/Duration 1 day/Duration 2 days/Duration 3 days/Duration >3 days/It interfered with the child’s usual activity/It did not interfere with the child’s usual activity/A phone or face-to-face consultation with his/her pediatrician regarding the adverse effect was required/No phone or face-to-face consultation with his/her pediatrician regarding the adverse effect was required.
24. Please mark which of the following adverse effects you observed after the administration of the vaccine [Malaise]: Duration <1 day/Duration 1 day/Duration 2 days/Duration 3 days/Duration >3 days/It interfered with the child’s usual activity/It did not interfere with the child’s usual activity/A phone or face-to-face consultation with his/her pediatrician regarding the adverse effect was required/No phone or face-to-face consultation with his/her pediatrician regarding the adverse effect was required.
25. Please mark which of the following adverse effects you observed after the administration of the vaccine [Shivering]: Duration <1 day/Duration 1 day/Duration 2 days/Duration 3 days/Duration >3 days/It interfered with the child’s usual activity/It did not interfere with the child’s usual activity/A phone or face-to-face consultation with his/her pediatrician regarding the adverse effect was required/No phone or face-to-face consultation with his/her pediatrician regarding the adverse effect was required.
26. Please mark which of the following adverse effects you observed after the administration of the vaccine [Low-grade fever (37.5-37.9ºC)]: Duration <1 day/Duration 1 day/Duration 2 days/Duration 3 days/Duration >3 days/It interfered with the child’s usual activity/It did not interfere with the child’s usual activity/A phone or face-to-face consultation with his/her paediatrician regarding the adverse effect was required/No phone or face-to-face consultation with his/her paediatrician regarding the adverse effect was required.
27. Please mark which of the following adverse effects you observed after the administration of the vaccine [Moderate fever (38-39ºC)]: Duration <1 day/Duration 1 day/Duration 2 days/Duration 3 days/Duration >3 days/It interfered with the child’s usual activity/It did not interfere with the child’s usual activity/A phone or face-to-face consultation with his/her paediatrician regarding the adverse effect was required/No phone or face-to-face consultation with his/her paediatrician regarding the adverse effect was required.
28. Please mark which of the following adverse effects you observed after the administration of the vaccine [High fever (>39ºC)]: Duration <1 day/Duration 1 day/Duration 2 days/Duration 3 days/Duration >3 days/It interfered with the child’s usual activity/It did not interfere with the child’s usual activity/A phone or face-to-face consultation with his/her paediatrician regarding the adverse effect was required/No phone or face-to-face consultation with his/her paediatrician regarding the adverse effect was required.
29. Indicate if your child has experienced any symptoms other than those described.
30. Did any of these symptoms require treatment? Yes/No.
31. If the answer to the above question was yes, indicate the treatment received and the duration in days.
32. Following your experience in vaccinating your child, would you recommend to your family members and friends that they vaccinate their children against the flu? Yes/No.
33. If the answer to the previous question is no, please explain your reason. Because the vaccine is not highly effective/Because of side effects/Because the influenza vaccine is not that important/Other.

Survey of parents/legal guardians of children aged 24 to 59 months vaccinated with LAIV.

1. How old is your child? 2 years/3 years/4 years.
2. Is the child a boy or girl? Girl/Boy.
3. Where was your child born? Spain/Fill in free text.
4. Was your child born prematurely? Yes/No.
5. How many brothers or sisters does your child have (excluding the child in)? None/1 sibling/2 siblings/>2 siblings.
6. Does your child have any chronic illnesses? Yes/No.
7. If yes, please specify what type.
8. How old are you? Under 20 years/20-29 years/30-39 years/40-49 years/Over 50 years.
9. What is your gender? Male/Female.
10. What is your country of birth? Spain/Fill in free text.
11. What is your level of education? No studies/Primary studies/Secondary studies/University studies.
12. Do you have any chronic illnesses? Yes/No.
13. If yes, please specify what type.
14. Did your child receive any more vaccines on the same day he/she received the flu vaccine? Yes/No.
15. Please mark which of the following adverse effects you observed after the administration of the vaccine [Headache]: Duration <1 day/Duration 1 day/Duration 2 days/Duration 3 days/Duration >3 days/It interfered with the child’s usual activity/It did not interfere with the child’s usual activity/A phone or face-to-face consultation with his/her paediatrician regarding the adverse effect was required/No phone or face-to-face consultation with his/her paediatrician regarding the adverse effect was required.
16. Please mark which of the following adverse effects you observed after the administration of the vaccine [Pain at the injection site]: Duration <1 day/Duration 1 day/Duration 2 days/Duration 3 days/Duration >3 days/It interfered with the child’s usual activity/It did not interfere with the child’s usual activity/A phone or face-to-face consultation with his/her paediatrician regarding the adverse effect was required/No phone or face-to-face consultation with his/her paediatrician regarding the adverse effect was required.
17. Please mark which of the following adverse effects you observed after the administration of the vaccine [Reddening at the injection site]: Duration <1 day/Duration 1 day/Duration 2 days/Duration 3 days/Duration >3 days/It interfered with the child’s usual activity/It did not interfere with the child’s usual activity/A phone or face-to-face consultation with his/her paediatrician regarding the adverse effect was required/No phone or face-to-face consultation with his/her paediatrician regarding the adverse effect was required.
18. Please mark which of the following adverse effects you observed after the administration of the vaccine [Inflammation at the injection site]: Duration <1 day/Duration 1 day/Duration 2 days/Duration 3 days/Duration >3 days/It interfered with the child’s usual activity/It did not interfere with the child’s usual activity/A phone or face-to-face consultation with his/her paediatrician regarding the adverse effect was required/No phone or face-to-face consultation with his/her paediatrician regarding the adverse effect was required.
19. Please mark which of the following adverse effects you observed after the administration of the vaccine [Induration at the injection site]: Duration <1 day/Duration 1 day/Duration 2 days/Duration 3 days/Duration >3 days/It interfered with the child’s usual activity/It did not interfere with the child’s usual activity/A phone or face-to-face consultation with his/her pediatrician regarding the adverse effect was required/No phone or face-to-face consultation with his/her paediatrician regarding the adverse effect was required.
20. Please mark which of the following adverse effects you observed after the administration of the vaccine [Muscle pain]: Duration <1 day/Duration 1 day/Duration 2 days/Duration 3 days/Duration >3 days/It interfered with the child’s usual activity/It did not interfere with the child’s usual activity/A phone or face-to-face consultation with his/her paediatrician regarding the adverse effect was required/No phone or face-to-face consultation with his/her paediatrician regarding the adverse effect was required.
21. Please mark which of the following adverse effects you observed after the administration of the vaccine [Low-grade fever (37.5-37.9ºC)]: Duration <1 day/Duration 1 day/Duration 2 days/Duration 3 days/Duration >3 days/It interfered with the child’s usual activity/It did not interfere with the child’s usual activity/A phone or face-to-face consultation with his/her paediatrician regarding the adverse effect was required/No phone or face-to-face consultation with his/her paediatrician regarding the adverse effect was required.
22. Please mark which of the following adverse effects you observed after the administration of the vaccine [Moderate fever (38-39ºC)]: Duration <1 day/Duration 1 day/Duration 2 days/Duration 3 days/Duration >3 days/It interfered with the child’s usual activity/It did not interfere with the child’s usual activity/A phone or face-to-face consultation with his/her paediatrician regarding the adverse effect was required/No phone or face-to-face consultation with his/her paediatrician regarding the adverse effect was required.
23. Please mark which of the following adverse effects you observed after the administration of the vaccine [High fever (>39ºC)]: Duration <1 day/Duration 1 day/Duration 2 days/Duration 3 days/Duration >3 days/It interfered with the child’s usual activity/It did not interfere with the child’s usual activity/A phone or face-to-face consultation with his/her paediatrician regarding the adverse effect was required/No phone or face-to-face consultation with his/her paediatrician regarding the adverse effect was required.
24. Please check any of the following adverse effects observed following the administration of the vaccine [Runny nose or nasal congestion]: Duration <1 day/Duration 1 day/Duration 2 days/Duration 3 days/Duration >3 days/It interfered with the child’s usual activity/It did not interfere with the child’s usual activity/A phone or face-to-face consultation with his/her paediatrician regarding the adverse effect was required/No phone or face-to-face consultation with his/her paediatrician regarding the adverse effect was required.
25. Please check any of the following adverse effects observed following the administration of the vaccine [Decreased appetite]: Duration <1 day/Duration 1 day/Duration 2 days/Duration 3 days/Duration >3 days/It interfered with the child’s usual activity/It did not interfere with the child’s usual activity/A phone or face-to-face consultation with his/her paediatrician regarding the adverse effect was required/No phone or face-to-face consultation with his/her paediatrician regarding the adverse effect was required.
26. Please check any of the following adverse effects observed following the administration of the vaccine [Malaise or decrease in energy]: Duration <1 day/Duration 1 day/Duration 2 days/Duration 3 days/Duration >3 days/It interfered with the child’s usual activity/It did not interfere with the child’s usual activity/A phone or face-to-face consultation with his/her paediatrician regarding the adverse effect was required/No phone or face-to-face consultation with his/her paediatrician regarding the adverse effect was required.
27. Please check any of the following adverse effects observed following the administration of the vaccine [Nosebleeds]: Duration <1 day/Duration 1 day/Duration 2 days/Duration 3 days/Duration >3 days/It interfered with the child’s usual activity/It did not interfere with the child’s usual activity/A phone or face-to-face consultation with his/her paediatrician regarding the adverse effect was required/No phone or face-to-face consultation with his/her paediatrician regarding the adverse effect was required.
28. Please check any of the following adverse effects observed following the administration of the vaccine [Skin rash]: Duration <1 day/Duration 1 day/Duration 2 days/Duration 3 days/Duration >3 days/It interfered with the child’s usual activity/It did not interfere with the child’s usual activity/A phone or face-to-face consultation with his/her paediatrician regarding the adverse effect was required/No phone or face-to-face consultation with his/her paediatrician regarding the adverse effect was required.
29. Indicate if your child has experienced any symptoms other than those described.
30. Did any of these symptoms require treatment? Yes/No.
31. If the answer to the above question was yes, indicate the treatment received and the duration in days.
32. Following your experience in vaccinating your child, would you recommend to your family members and friends that they vaccinate their children against the flu? Yes/No.
33. If the answer to the previous question is no, please explain your reason. Because the vaccine is not highly effective/Because of side effects/Because the influenza vaccine is not that important/Other.
